# Anti‐inflammatory and immunoregulatory effects of colistin sulphate on human PBMCs

**DOI:** 10.1111/jcmm.18322

**Published:** 2024-04-25

**Authors:** Huiling Chen, Tianli Yang, Yiran Xu, Beibei Liang, Xianyong Liu, Yun Cai

**Affiliations:** ^1^ Department of Pharmacy Center of Medicine Clinical Research, Medical Supplies Center, PLA General Hospital Beijing China; ^2^ Department of Pharmacy Zigong Fourth People's Hospital Zigong China; ^3^ Medical School of Chinese PLA Graduate School of Chinese PLA General Hospital Beijing China; ^4^ The Second Naval Hospital of Southern Theater Command of PLA Sanya China; ^5^ Department of Thoracic Surgery The First Medical Center, PLA General Hospital Beijing China

**Keywords:** anti‐inflammatory, colistin sulphate, cytokine–cytokine receptor interaction, immunoregulatory effects, PBMCs

## Abstract

In previous studies, CST has been identified as having an immunostimulatory effect on *Caenorhabditis elegans* and macrophage of rats. Here, we further investigated its immunomodulatory effects on human peripheral blood mononuclear cells (PBMCs). LPS‐stimulated PBMCs inflammatory model was established. Flow cytometry was applied to measure phagocytosis of PBMCs. Cytokine mRNA and protein expression levels of LPS‐stimulated PBMCs with or without CST were measured by qRT‐PCR and ELISA. The transcriptomic profile of CST‐treated PBMCs was investigated by RNA‐sequencing. Gene Ontology (GO) and Kyoto Encylopedia of Genes and Genomes (KEGG) were applied to find potential signalling pathways. PBMCs showed a significant increase in phagocytic activity at 6 h after being incubated with CST at the concentration of 10 μg/mL. In the presence of LPS, CST maintained and promoted the expression of TNF‐α and chemokine CCL24. The content of pro‐inflammatory cytokines, such as IL‐1β, IL‐6 and IFN‐γ, which were released from LPS‐stimulated PBMCs, was reduced by CST at 6 h. Anti‐inflammatory cytokines, such as IL‐4, IL‐13 and TGF‐β1, were significantly increased by CST at 24 h. A total of 277 differentially expressed immune‐related genes (DEIRGs) were detected and cytokine‐cytokine receptor interaction was highly enriched. CST presented obvious anti‐inflammatory and immunoregulatory effects in LPS‐induced PBMCs inflammatory model not only by improving the ability of PBMCs to clear pathogens but also by decreasing pro‐inflammatory cytokines and increasing anti‐inflammatory cytokines. And the mechanism may be related to cytokine‐cytokine receptor interaction.

## 1 INTRODUCTION

Increased antimicrobial resistance (AMR) is a threat to modern medicine and requires global action. Drug‐resistant microorganisms have resulted in higher infection morbidity and mortality, placing a greater burden on health systems.^1^, ^2^ New developed antimicrobials have successfully solved this problem for a long time. However, since the last decades of 20th century, the drug discovery failed to keep up with the increase of drug‐resistant microorganisms despite the increased efforts to develop new antibacterial agents. Drugs approved over the past few years were scarce, which didn't significantly improve the existing dilemma.^3^, ^4^, ^5^ Thus, more and more non‐traditional approaches aiming to influence the disease itself instead of only inhibiting or killing pathogens have been becoming popularity.^6^

Nowadays, researchers expect to minimise or overcome resistance by enhancing host immune system instead of only using antibiotics. It was the main strategy of the third age of antimicrobial therapy, which combined immunotherapy and antibiotics therapy.^7^ As we all know, the immune system of host invaded by microbes plays a crucial role in protecting from infection disease. Host could rapidly initiate a strong inflammatory immune response to phagocytose and eliminate the pathogens, which began with the pattern‐recognition receptors (PRRs) recognising and binding pathogen‐associated molecular patterns (PAMPs) expressed by microbes or damage‐associated molecular patterns (DAMPs) released from damaged tissue, respectively.^6^, ^8^ These PRRs are expressed on a variety of immune cells, and major including Toll‐like receptors (TLRs), C‐type lectin receptors (CLRs), nucleotide‐binding oligomerization domain (NOD)‐like receptors (NLRs), retinoic‐acid‐inducible gene‐I (RIG‐I)‐like receptors (RLRs) and receptor for advanced glycation end products (RAGE).^9^ Typical transcription programme of PRRs involved activating signal transduction pathways of key transcription factors including NF‐κB, activator protein1 (AP‐1), interferon regulatory factors (IRFs) and nuclear factor of activated T cells (NFAT).^10^ In response, pathogens could successfully survive, replicate and eventually promote infection by manipulating host defence mechanisms, such as the NF‐κB pathway and the cell death pathway (such apoptosis).^11^, ^12^, ^13^, ^14^, ^15^ Host immune dysregulation was the fundamental molecular mechanism of the pathophysiological basis of many infectious diseases, which generally led to severe sepsis, septic shock, multiple organ failure and death.^16^, ^17^, ^18^, ^19^ And a substantial proportion of survivors from critical illnesses such as sepsis/septic shock often suffered long‐term severe dysfunctional immunity (termed chronic critical illness (CCI)) including persistent inflammation, immunosuppression and catabolism syndrome (PICS), which were associated with increased mortality.^8^, ^20^, ^21^ Therefore, it is greatly related to mortality of critical illness whether it is able to modulate the immune system in the early stage to reduce the conversion rate.

In recent years, many studies have found that lots of antimicrobials have potentially beneficial non‐anti‐infective properties such as anti‐inflammatory and immunomodulatory activities and therapeutic effect.^22^, ^23^ Repurposing such drugs in immune disordered patients is a very promising approach and may fill the antibiotic discovery void with several advantages such as known safety and pharmacokinetic characteristics and low time and economic costs for other therapeutic applications. Colistin (also known as polymyxin E) was a polypeptide antibiotic first found in 1947 with activity against most Gram‐negative bacteria. However, on account of the adverse events of the nephrotoxicity and neurotoxicity, coupled with the discovery and approval of novel highly effective and low‐toxic antibiotics such as aminoglycosides, quinolones and β‐lactams, colistin was once abandoned clinically in the 1970s.^24^, ^25^ With the increasing emergence and prevalence of multidrug‐resistant (MDR) Gram‐negative bacteria, the use of colistin was seriously reconsidered as a last‐resort therapeutic option.^26^, ^27^, ^28^ In recent years, studies have identified that colistin sulphate (CST) provided a non‐anti‐infective protection for animal cells to fight bacterial infections mainly through enhancing PMK‐1/p38 MAPK pathway to mediate immune responses.^29^, ^30^ Here, we tend to further investigate the immunomodulatory effects and related mechanisms of CST in human PBMCs.

## 2 MATERIALS AND METHODS

### 2.1 Reagents

CST (MCE, HY‐A0089) was dissolved in RPMI1640 medium (CORNING, 10‐040‐CV) with 10% foetal bovine serum (FBS) (Gibco, 10,099,141) at a concentration of 4 mg/mL as a stock solution. Several final concentrations of CST (5, 10, 20 and 40 μg/mL) were used in the following experiments to decide the best optimum concentration.

### 2.2 PBMCs isolation

Venous blood was taken from the healthy volunteers, and the samples were EDTA‐anticoagulated. The anticoagulated blood was treated by Ficoll–Hypaque density gradient centrifugation (Solarbio, P9011), and the PBMCs were separated. PBMCs were washed and resuspended in RPMI 1640 medium, supplemented with 10% FBS, at a concentration of 1.5 × 10^6^ cells/ml for investigation. Ethical approval and informed consent for each donor were granted by the ethics committee of Chinese PLA General Hospital (S2021‐046‐01).

### 2.3 Phagocytosis of fluorescent latex beads

The PBMCs (1.5 × 10^6^ cells/ml) were pre‐cultured with several concentrations of CST (5, 10, 20, 40 μg/mL) in humidified air with 5% CO_2_ for 3, 6, 12, 24 and 48 h. After pre‐incubation, the PBMCs were washed three times by Dulbecco's phosphate buffered saline (D‐PBS) (Solarbio, D1040). Then, each culture was mixed with 1% (v/v) fluorescent latex particles (1.0 μm in diameter) (Sigma‐Aldrich, L4655) and incubated at 37°C for 2 h. The single‐cell suspensions were washed with D‐PBS before and after staining with cell surface markers. Cells were incubated with multiple fluorescently labelled monoclonal antibodies 15 min to delineate phagocytosis of PBMCs. The following antibodies were utilised in 100 μL PBS: PerCP anti‐human CD45 (Caprico Biotechnologies, S045PC04) and PE anti‐human CD11b (Biolegend, 301,306). Fluorescences were evaluated by flow cytometer (Beckman Coulter, DxFLEX), and phagocytic activity was expressed as the percentage of cells from the total number of viable PBMCs that performed phagocytosis.

### 2.4 Inflammatory cytokine measurement

PBMC cells at a concentration of 1.5 × 10^6^ cells/ml in polystyrene round‐bottom tube (FALCON, 352054) were separately incubated with culture medium or 10 μg/mL CST, 0.1 μg/mL LPS (Sigma‐Aldrich, L2880), a combination of CST with LPS or CST pre‐incubation for 6 h followed by LPS. After 6 h and 24 h, the cells and cell culture supernatants were separately collected and stored at −80°C for further analysis. Inflammatory cytokine production was measured in the supernatants of PBMCs cultured in the conditions defined above. ELISA kits from MLBio were utilised to quantify the following cytokine concentrations following the manufacturer's instructions: IL‐1β (ml027417), IL‐6 (ml028583), TNF‐α (ml022565), IFN‐γ (ml027464), IL‐4 (ml028585), IL‐10 (ml028605), IL‐13 (ml027429) and TGF‐β1 (ml022522). These cytokines are indicative of the inflammatory function of PBMC.

### 2.5 RNA extraction and real‐time qRT‐PCR

PBMCs were treated under different conditions defined above, and total RNA was isolated using the Direct‐zol RNA Miniprep plus kits (ZYMO RESEARCH, R2072) and TRI Reagent (Invitrogen, 15,596,026) according to the manufacturer's protocol. RNA was quantified, and its purity was validated by NanoDrop (Thermo Fisher Scientific). Complementary DNA (cDNA) was synthesized from total RNA with the First Stand cDNA Synthesis Kit (NEB, E6560S). Quantitative real‐time reverse transcription‐polymerase chain reaction (qRT‐PCR) was carried out using PowerUP SYBR Green Master Mix (Applied Biosystems, A25742). The relative expressions of mRNA for all target genes were determined using the $2^{-\Delta \Delta C_{t}}$ method. Target‐gene transcripts in each sample were normalized to β‐actin. Inflammatory cytokine‐related primer sequences used for the qRT‐PCR analysis are listed in Table 1.

**TABLE 1:** List of inflammatory cytokine‐related primer sequences used for the qRT‐PCR.

Gene ID	Primer (5′ → 3′)
IL1β‐F	TTCGAGGCACAAGGCACAA
IL1β‐R	TGGCTGCTTCAGACACTTGAG
IL6‐F	CTCCTTCTCCACAAGCGCC
IL6‐R	GAAGGCAGCAGGCAACAC
TNFα‐F	CAAGGACAGCAGAGGACCAG
TNFα‐R	AGAGGCTGAGGAACAAGCAC
IFNγ‐F	GGCTTTTCAGCTCTGCATCG
IFNγ‐R	CTGGGATGCTCTTCGACCTC
IL4‐F	CCAACTGCTTCCCCCTCTG
IL4‐R	TCTGTTACGGTCAACTCGGTG
IL10‐F	AATAAGGTTTCTCAAGGGGCT
IL10‐R	AGAACCAAGACCCAGACATCAA
IL13‐F	CCTCATGGCGCTTTTGTTGAC
IL13‐R	TCTGGTTCTGGGTGATGTTGA
TGFβ1‐F	CAATTCCTGGCGATACCTCAG
TGFβ1‐R	GCACAACTCCGGTGACATCAA
β‐actin‐F	AGCGGGAAATCGTGCGTG
β‐actin‐R	CAGGGTACATGGTGGTGCC

### 2.6 Bulk RNA sequencing and differentially gene expression analysis

The PBMCs (1.5 × 10^6^ cells/ml) were treated with culture medium or 10 μg/mL CST in the presence or absence of LPS for 24 h. Then cells were stored in RNA stabilization solution (Invitrogen, AM7020) for subsequent RNA extraction and quality assessment. Purified RNA (1000 ng) from each sample was used as the starting template for library construction. Libraries were prepared using KAPA Hyper Prep Kits (KAPA, KK8504) for Illumina. Fragment Analyser 1.0.2.9 was used to check the fragment size of the library (between 300 and 700 bp), and the effective concentration (>10 nmol/L) was accurately quantified by Q‐PCR to ensure the quality of the library. Paired‐end sequencing (150 bp for each read) was performed based on Illumina Novaseq system with at least 16 million reads for each sample. Quality of sequenced reads was assessed using FastQC, and reads were filtered and trimmed by Trimmomatic v0.39. Reads were mapped to the reference genomes of human (hg19) using STAR 2.7.5c with default setting. We used StringTie (v2.1.7) for gene quantification and obtained the standardised gene‐level fragments per kilobase per million mapped fragments (FPKM) value. Normalisation of differentially expressed genes (DEGs) between control and treatments was performed using an R package, DESeq2. Genes with a *p* value < 0.05 and a FoldChange (FC) ≥ |1.2| were considered DEGs. Heatmap visualisations used log2 counts per million (log2cpm). Data sets were clustered by 1 minus Pearson's correlation coefficient. Enriched genes were extracted and shown in volcano plot drawn by R package Enhanced Volcano v1.5.4. Gene Ontology (GO) enrichment analysis was performed using TopGO (v2.24.0). The KEGG pathway analysis performed the top 40 enriched signalling pathways.

### 2.7 Analysis of differentially expressed immune‐related genes (DEIRGs)

To investigate the immune‐related genes affected by CST, the sequencing data of DEGs was further analysed. The immune‐related genes (IRGs) list was obtained from ImmPort database (https://www.immport.org/shared/) including cytokine, cytokine receptor, interleukin (IL), interleukin receptor, interferon, interferon receptor, tumour necrosis factor (TNF) family member, TNF family member receptor, transforming growth factor (TGF‐b) family member, TGF‐b family member receptor, chemokine, chemokine receptor, T cell receptor (TCR) signalling pathway, breakpoint cluster region (BCR) signalling pathway, natural killer cell, antigen processing and presentation, or antimicrobial. We finally extracted 2499 IRGs for further analysis.

### 2.8 Statistical analysis

The statistical methods related to each figure are outlined in the figure legend. Results are presented as means with standard error of mean (SEM) to indicate the variation within each experiment. Statistics analysis was performed in Excel, GraphPad Prism. Shapiro–Wilk test was used to check normality of the data. One‐way analysis of variance (ANOVA) was used for the comparison among groups followed by the Tukey's honestly significant difference test (Tukey's HSD). Differences are determined to be statistically significant when a *p* value < 0.05 was attained.

## 3 RESULTS

### 3.1 CST increased phagocytosis of PBMCs

**FIGURE 1:**
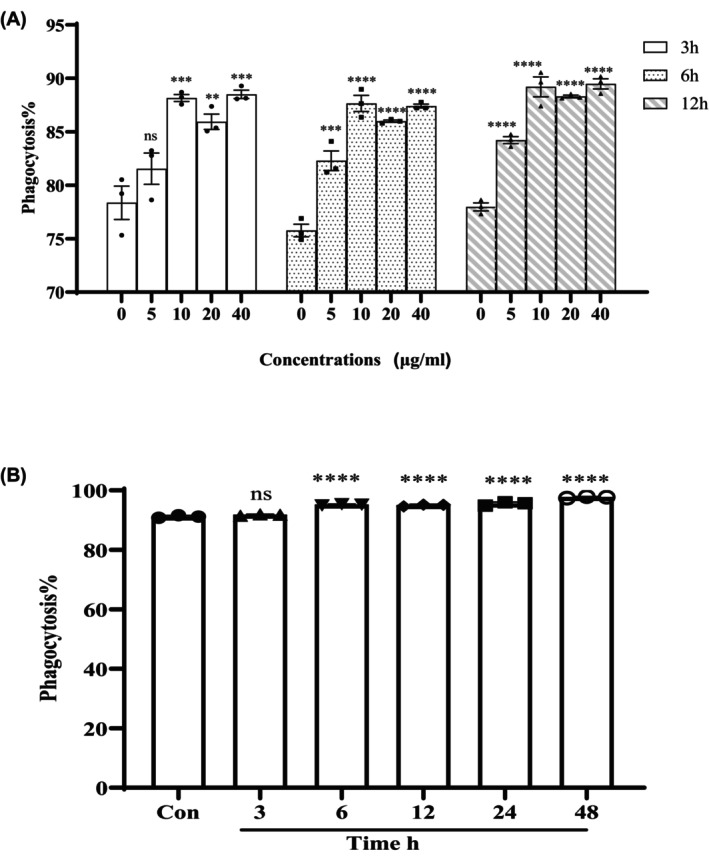
Effects of CST on phagocytosis of PBMCs. (A) Effects of Control (0) and varying concentrations of CST (5, 10, 20 and 40 μg/mL) on the phagocytosis of fluorescent latex beads. PBMCs from normal volunteers were incubated with CST for 3, 6 and 12 h for phagocytosis assay. (B) Time‐dependent effects of CST (10 μg/mL) on the phagocytosis of fluorescent latex beads by PBMCs. PBMCs from normal volunteers were incubated with CST for 0–48 h for phagocytosis assay. Data show percentage of PBMCs positive for phagocytosis determined and are expressed as mean ± SEM and range of three separate experiments. Statistical significance was versus with control (0) and was indicated by *p < 0.05, **p < 0.01, ***p < 0.001; ****p < 0.0001; ns, not statistically significant.

Treatment with 10 μg/mL of CST significantly increased the percentage of PBMCs that had phagocytosed fluorescent latex beads, compared with untreated PBMCs (Figure 1A). No obvious increasement was observed in phagocytosis with higher doses of CST beyond 10 μg/mL (Figure 1A). Considering the toxicity of CST, 10 μg/mL was chosen as the optimal concentration.

The effects of phagocytosis were also dependent on the length of incubation time with CST. PBMCs incubated with CST for 6–24 h showed similar increased phagocytosis compared with control and 3 h incubation (Figure 1B). Even though the phagocytosis of PMBCs achieved the highest at 48 h incubation, long‐term incubation with CST may also face the problem of increased toxicity.

### 3.2 CST decreased LPS‐activated pro‐inflammatory cytokine of PBMCs at gene and protein levels at 6 h

**FIGURE 2:**
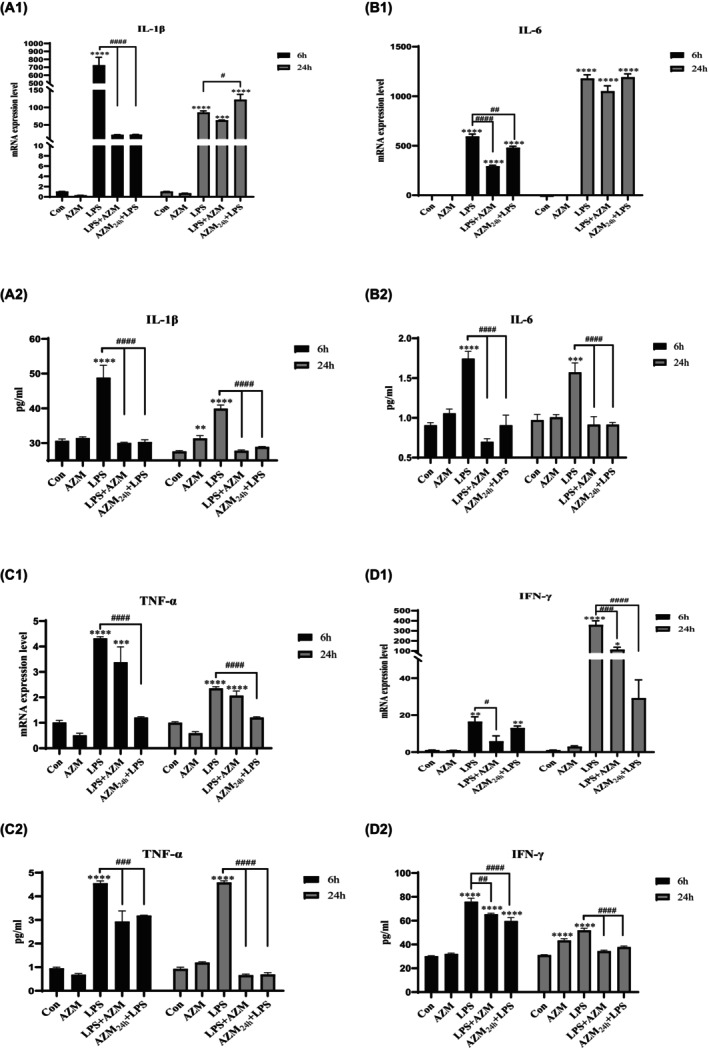
Effect of CST on pro‐inflammatory cytokines gene expression and protein production levels in unstimulated and LPS‐activated PBMCs. PBMCs were incubated with CST in the presence or absence of LPS for 6 and 24 h, respectively. (A_1_–D_1_) qRT‐PCR was performed to measure gene expression levels of IL‐1β (A_1_), IL‐6 (B_1_), TNF‐α (C_1_), IFN‐γ (D_1_) after normalization to β‐Actin. Each column represents the fold‐change of mRNA expression levels comparing either unstimulated control or LPS‐stimulated group. (A_2_–D_2_) PBMCs were incubated with CST for 0 h or 6 h followed by LPS stimulation for another 6 h and 24 h. Then, the cell culture supernatants were collected and assayed by ELISA for protein levels of IL‐1β (A2), IL‐6 (B2), TNF‐α (C2), IFN‐γ (D2) respectively. Data are expressed as mean ± SEM, (*) means treated groups versus unstimulated control, (#) means CST‐treated groups in the presence of LPS versus LPS mono‐stimulation group. Statistical significance was indicated by */#*p* < 0.05, **/##*p* < 0.01, ***/###*p* < 0.001; ****/####*p* < 0.0001.

As shown in Figure 2A_1_–D_1_ and Figure 2A_2_–D_2_, stimulation with CST alone showed little effect on pro‐inflammatory cytokines, except IFN‐γ. At 6 h, IFN‐γ protein levels were increased in CST mono‐stimulated PBMCs when compared with unstimulated control (Figure 2D_2_; *p* < 0.01). The LPS differentially activated the expression of all pro‐inflammatory cytokines in PBMCs while CST decreased LPS‐activated gene and protein expression of the pro‐inflammatory cytokines IL‐1β, IL‐6, TNF‐α and IFN‐γ over time.

LPS enhanced IL‐1β gene expression levels up to 729.9‐fold after 6 h of incubation (Figure 2A_1_; *p* < 0.0001), and this effect attenuated and was maintained at 85.2‐fold change after 24 h (*p* < 0.0001). However, CST statistically reduced IL‐1β gene expression level in the presence of LPS at 6 h compared with LPS group (*p* < 0.0001). Similar trend was found in IL‐6 gene expression (both at 6 and 24 h) and IFN‐γ gene expression (at 6 h) in LPS‐activated PBMCs (Figure 2B_1_; *p* < 0.0001; Figure 2D_1_; *p* < 0.01). The exception is that CST didn't decrease the expression of TNF‐α induced by LPS (Figure 2C_1_).

To determine whether protein levels of IL‐1β, IL‐6, TNF‐α, IFN‐γ correlate with respective gene expression level, cell culture supernatant was collected after 6 h and 24 h of stimulation with LPS to measure cytokine protein levels in unstimulated, LPS‐activated and CST‐treated PBMCs.

The changes of protein levels were basically consistent with the genetic changes. CST statistically decreased the release of LPS‐activated IL‐1β, IL‐6 and IFN‐γ when compared to LPS group (Figure 2A_2_; *p* < 0.0001; Figure 2B_2_; *p* < 0.0001; Figure 2D_2_; *p* < 0.0001), which was consistent with the changes of gene expression. In the presence of LPS, CST showed no decreasing effect on TNF‐α protein production at 6 h, but at 24 h, an obvious reduction was observed, which was different with gene expression (Figure 2C_2_; *p* < 0.0001). Further, CST directly co‐incubated with LPS showed a stronger effect in reducing LPS‐activated pro‐inflammatory cytokine than pre‐incubated with PBMCs.

### 3.3 CST increased LPS‐activated anti‐inflammatory cytokine at gene and protein levels at 24 h

**FIGURE 3:**
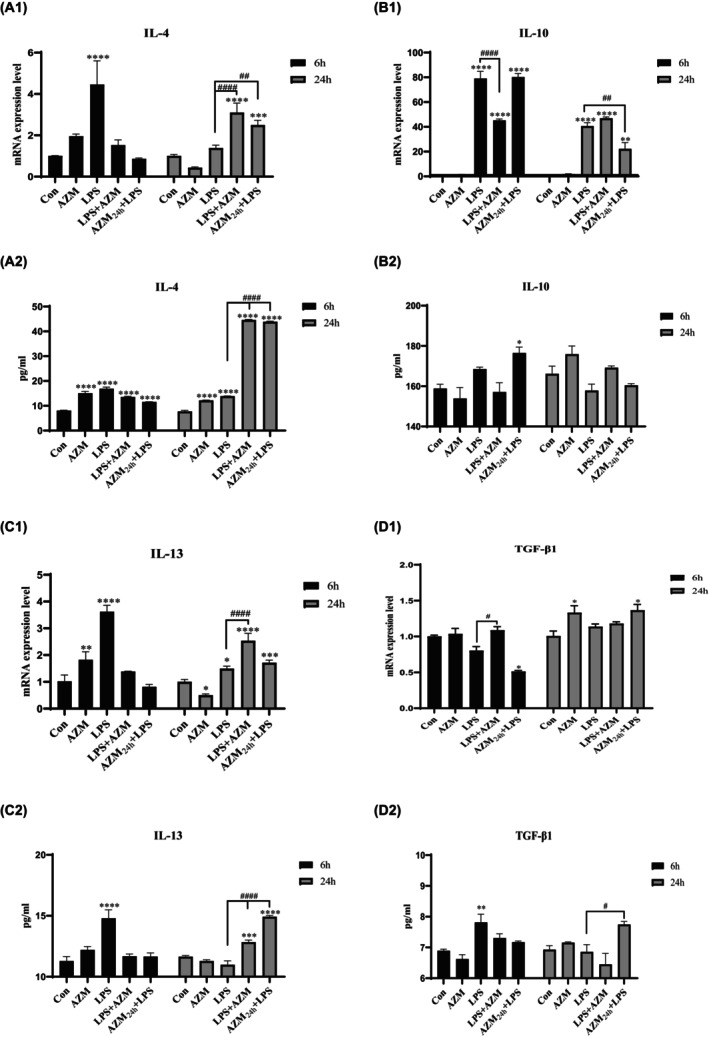
Effect of CST on anti‐inflammatory cytokines gene expression and protein production levels in unstimulated and LPS‐activated PBMCs. PBMCs were incubated with CST in the presence or absence of LPS for 6 and 24 h, respectively. (A_1_–D_1_) qRT‐PCR was performed to measure gene expression levels of IL‐4 (A_1_), IL‐10 (B_1_), IL‐13 (C_1_), TGF‐β1 (D_1_) after normalization to β‐Actin. Each column represents the fold‐change of mRNA expression levels comparing either unstimulated control or LPS‐stimulated group. (A_2_–D_2_) PBMCs were incubated with CST for 0 h or 6 h followed by LPS stimulation for another 6 and 24 h. Then, the cell culture supernatants were collected and assayed by ELISA for protein levels of IL‐4 (A_2_), IL‐10 (B_2_), IL‐13 (C_2_) and TGF‐β1 (D_2_) respectively. Data are expressed as mean ± SEM, (*) means treated groups versus unstimulated control, (#) means CST‐treated groups in the presence of LPS versus LPS mono‐stimulation group. Statistical significance was indicated by */#*p* < 0.05, **/##*p* < 0.01, ***/###*p* < 0.001; ****/####*p* < 0.0001.

At 6 h, LPS differentially activated the expression of anti‐inflammatory cytokines IL‐4, IL10 and IL‐13, which were decreased by CST. The level of anti‐inflammatory cytokines in LPS group at 24 h eased back from 6 h, and CST obviously increased the gene levels of anti‐inflammatory cytokines, except IL‐10 (Figure 3A_1_–D_1_).

Mono‐incubation with CST increased TGF‐β1 gene expression at 24 h when compared to unstimulated control (Figure 3D_1_; *p* < 0.01). Moreover, CST increased TGF‐β1 gene expression both at 6 h and 24 h when compared to LPS‐activated group (Figure 3D_1_; *p* < 0.01, *p* < 0.001). Similarly, IL‐4 and IL‐13 gene expression levels were significantly enhanced by CST at 24 h when compared to LPS‐activated group (Figure 3A_1_; *p* < 0.0001 and Figure 3C_1_; *p* < 0.001). However, CST on IL‐10 increase in the presence of LPS was not obvious (Figure 3B_1_).

Consistent with the results of gene expression, IL‐4, IL‐13 and TGF‐β1 protein levels were increased by CST in LPS‐activated PBMCs when compared to LPS group at 24 h (Figure 3A_2_; *p* < 0.0001; Figure 3C_2_; *p* < 0.0001; Figure 3D_2_; *p* < 0.0001). While in the presence of LPS, IL‐10 protein level was increased by CST when compared to LPS group at 6 and 24 h (Figure 3B_2_), even if the change in gene expression level was not obvious. Moreover, CST directly co‐incubated with LPS increased the expression level of anti‐inflammatory cytokines much more than pre‐incubation with CST followed by LPS stimulation.

### 3.4 DEGs significantly affected by CST in LPS‐activated PBMCs

**FIGURE 4:**
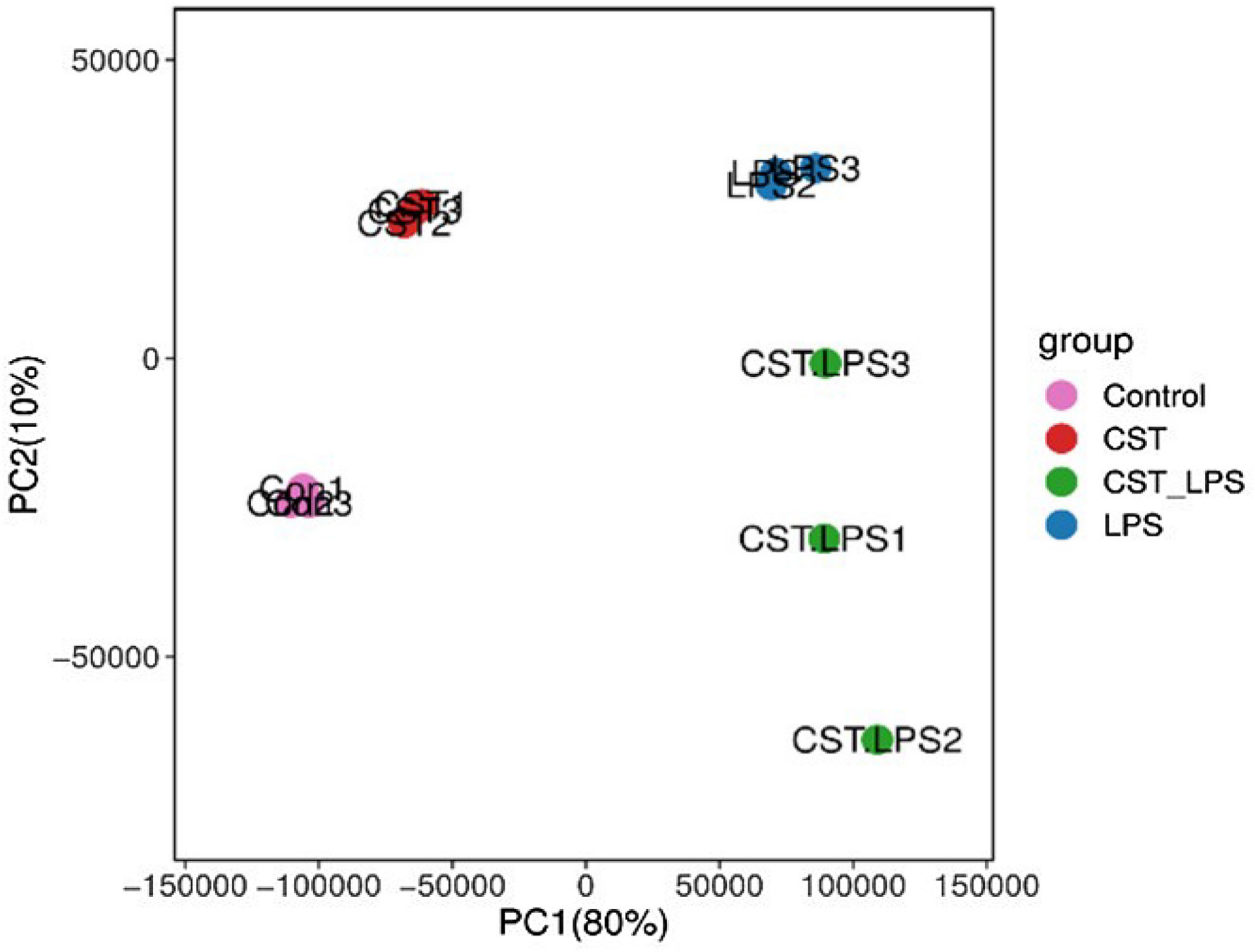
Principal component analysis (PCA). Each principal component (PC) on the y‐axis is plotted against the variance it contributes within the dataset on the x‐axis, the horizontal line indicates an arbitrary threshold for PCs that contribute a significant proportion of the variance. PCA was performed in R using normalized data from the DESeq2 analysis.

The principal component analysis (PCA) results (Figure 4) showed that samples clustering within groups, indicating that genetic variation of inter‐sample was close.

**FIGURE 5:**
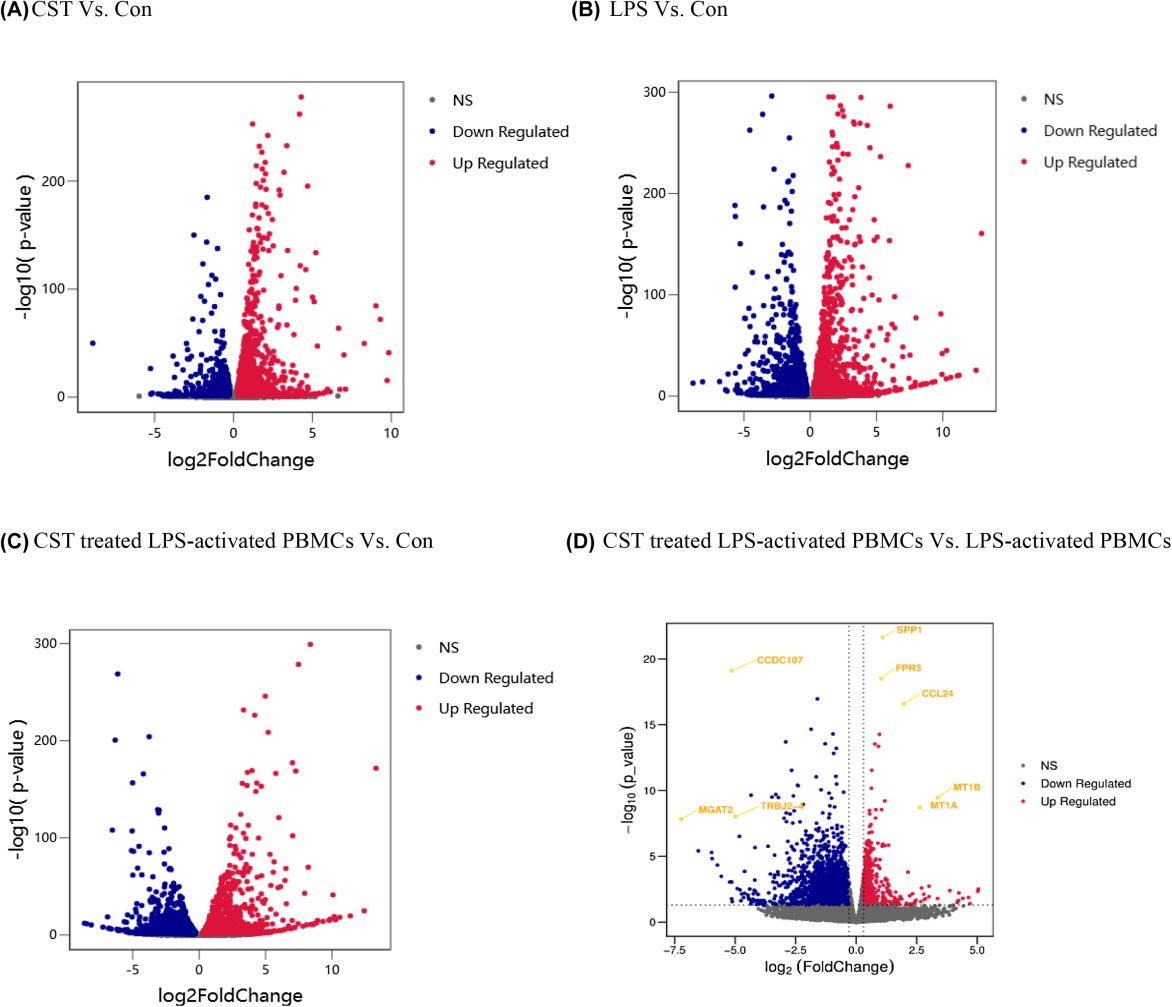
Volcano plot the differentially expressed genes (DEGs). Volcano plot showing upregulated (right) and downregulated (left) genes of PBMCs after CST treatment and LPS stimulation. Each dot stands for a gene. The x‐axis is calculated as $\mathrm{lo}g_{2}\left(\text{FoldChange}\right)$, and y‐axis is calculated as $-\mathrm{lo}g_{10}\left(p-\text{value}\right)$. Differentially expressed genes (DEGs) are marked in red (p<0.05 and Fold Change≥1.2) and in blue (p<0.05 and Fold Change ≤−1.2).

Significant and non‐significant genes compared between groups were plotted as volcano plots, as shown in Figure 5A–D. The significantly regulated genes are shown outside the dotted lines (with FoldChange ≤−1.2 and ≥1.2; *p*‐value < 0.05). After compared, we finally identified 3965 DEGs affected by CST in LPS‐activated PBMCs including 1372 upregulated genes and 2593 downregulated genes (Figure 5D). After multiple comparisons adjusting for *p* value, the most significantly changed genes (with lowest adjusted *p*‐value and highest fold change) were selected from among all the DEGs and marked in the volcano plot. Five genes were highly upregulated by CST in LPS‐activated PBMCs: Secreted phosphoprotein 1 (SPP1; padj = 3.20 × 10^−18^; log2 FC = 1.09) and Formyl peptide receptor 3 (FPR3; padj = 1.40 × 10^−15^; log2 FC = 1.04), C‐C motif chemokine ligand 24 (CCL24; padj = 7.36 × 10^−14^; log2 FC = 1.96), metallothionein 1B (MT1B; padj = 1.57 × 10^−7^; log2 FC = 3.37) and metallothionein 1A (MT1A; padj = 7.16 × 10^−7^; log2 FC = 2.63). Three genes were highly downregulated by CST in LPS‐activated PBMCs: Coiled‐coil domain containing 107 (CCDC107; padj = 5.38 × 10^−16^; log2 FC = ‐5.14), alpha‐1,6‐mannosyl‐glycoprotein 2‐beta‐N‐acetylglucosaminyltransferase (MGAT2; padj = 4.01 × 10^−6^; log2 FC = ‐7.22) and T cell receptor beta joining 2–4 (TRBJ2‐4; padj = 2.77 × 10^−6^; log2 FC = −4.98). And these significantly changed DEGs related to immunity were SPP1, FPR3, CCL24 and TRBJ2‐4.

### 3.5 DEIRGs Between CST treated LPS‐activated PBMCs and LPS‐activated PBMCs

**FIGURE 6:**
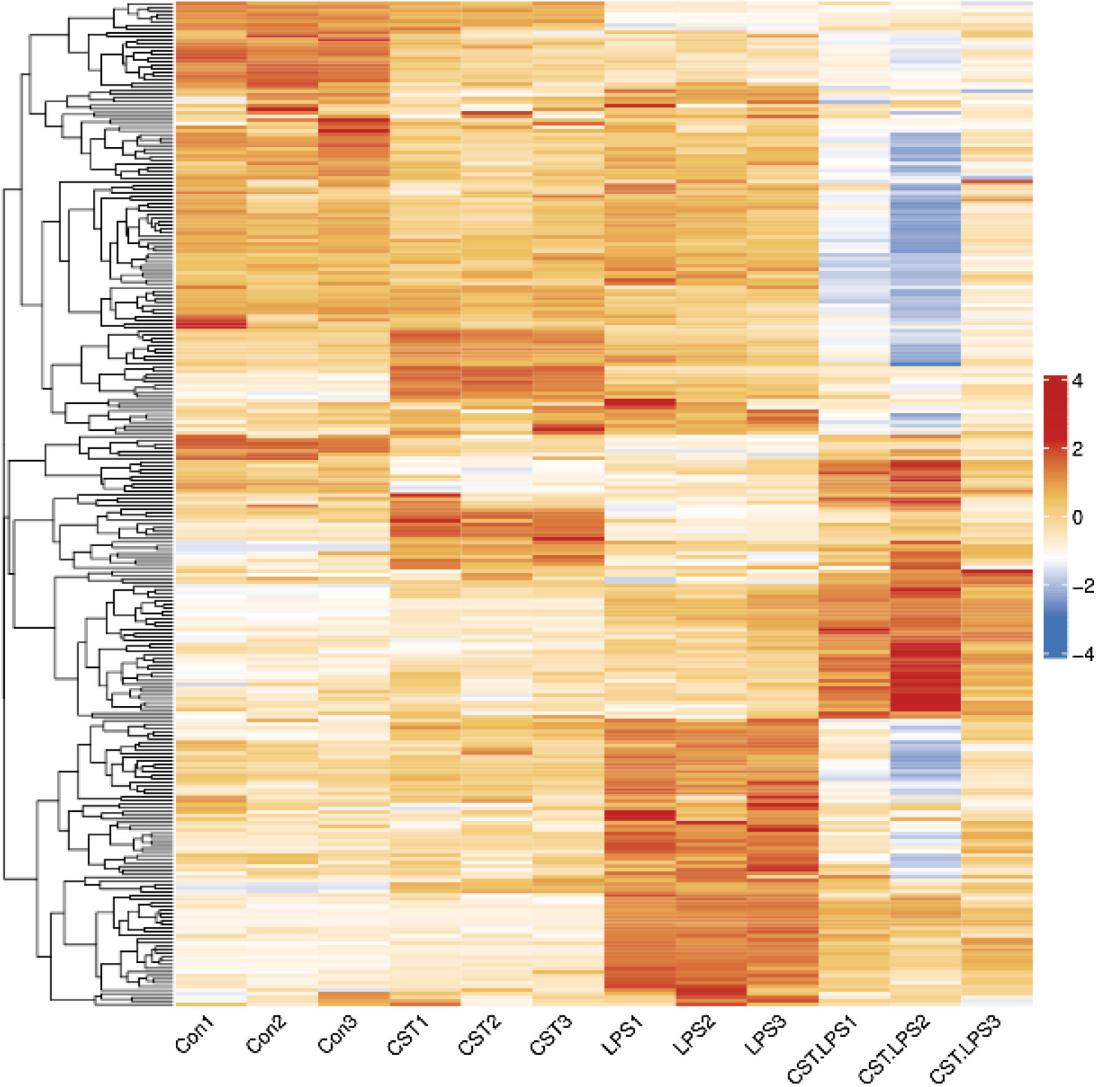
Heatmap of DEIRGs. Heatmap for DEIRGs. The 12 samples: the unstimulated PBMCs, LPS stimulated/vehicle treated, CST/vehicle treated and CST treated LPS‐activated PBMCs. The map was generated by broad Morpheus software. Heatmap visualisations used fragments per kilobase of transcript per million (FPKMS) and log2 FoldChange (log2FC). Data sets were hierarchically clustered using one minus Pearson correlation coefficient.

To screen out IRGs from the DEGs, we further selected the intersection of 3965 DEGs and 2499 IRGs and finally obtained 277 DEIRGs. The main clusters of DEIRGs expression levels in all treatment conditions mentioned were investigated and displayed in a heatmap (Figure 6). The top 20 DEIRGs based on an adjusted *p*‐value (padj) are presented in Table 2, and most of those genes are categorised as antimicrobials, chemokines (receptors), cytokines (receptors) and TCR signalling pathway. CST downregulated LPS‐activated DEIRGs including tumour necrosis factor ligand superfamily member 18 (TNFSF18); ADRM1 26S proteasome ubiquitin receptor (ADRM1), which participated in the antigen recognition; TCR or BCR signalling pathway‐related genes such as T cell receptor beta joining2–3, 2–4, 2–7 (TRBJ2‐3, TRBJ2‐4, TRBJ2‐7); p21 (RAC1) activated kinase 4 (PAK4); delta‐like canonical Notch ligand 4 (DLL4) and immunoglobulin lambda variable 7–46 (IGLV7‐46). Some other inflammatory response and immune response‐related genes were upregulated, such as secreted phosphoprotein 1 (SPP1), C‐C motif chemokine ligand 24 (CCL24), and alpha‐2‐macroglobulin (A2M) were upregulated in CST‐treated PBMCs.

**TABLE 2:** List of the top 20 differentially expressed immune‐related genes.

DEIRGs	Log(FC)	baseMean	padj	Category
SPP1	1.09	4182.29	3.20E‐18	Cytokines, cytokine receptors
FPR3	1.04	1908.47	1.40E‐15	Chemokine receptors
CCL24	1.96	540.61	7.36E‐14	Antimicrobials, chemokines, cytokines
A2M	1.18	263.35	2.60E‐07	Antimicrobials
TRBJ2‐4	−4.98	22.39	2.77E‐06	TCR signalling pathway
PAK4	−1.92	140.64	4.74E‐05	TCR signalling pathway
DLL4	−1.82	48.92	5.01E‐05	TCR signalling pathway
TRBJ2‐3	−2.55	21.40	0.000318	TCR signalling pathway
ADRM1	−1.41	506.52	0.000796	Antigen processing and presentation
TFR2	−1.18	76.40	0.001158	Antimicrobials
IGLV7‐46	−1.39	37.99	0.002103	BCR signalling pathway
DUOX2	−1.15	57.70	0.003965	Antimicrobials
PLXNA1	−1.34	434.71	0.004208	Chemokine receptors, cytokine receptors
TRBJ2‐7	−2.25	58.54	0.006143	TCR signalling pathway
PLXNB2	−1.37	2852.43	0.006964	Chemokine receptors, cytokine receptors
TYMP	−1.17	3349.17	0.007243	Chemokines, cytokines
BCL3	−1.17	1222.28	0.007268	Antimicrobials
UNC93B1	−1.24	660.67	0.007585	Antimicrobials
ESRRA	−1.52	277.47	0.011283	Cytokine receptors
TNFRSF18	−2.18	106.41	0.011763	Cytokine receptors, TNF family members receptors

Abbreviations: DIRGs, differentially expressed immune‐related genes; FC, fold change; padj, adjusted *p*‐value.

### 3.6 Functional enrichment analysis of DEIRGs in CST treated LPS‐activated PBMCs and LPS‐activated PBMCs

**FIGURE 7:**
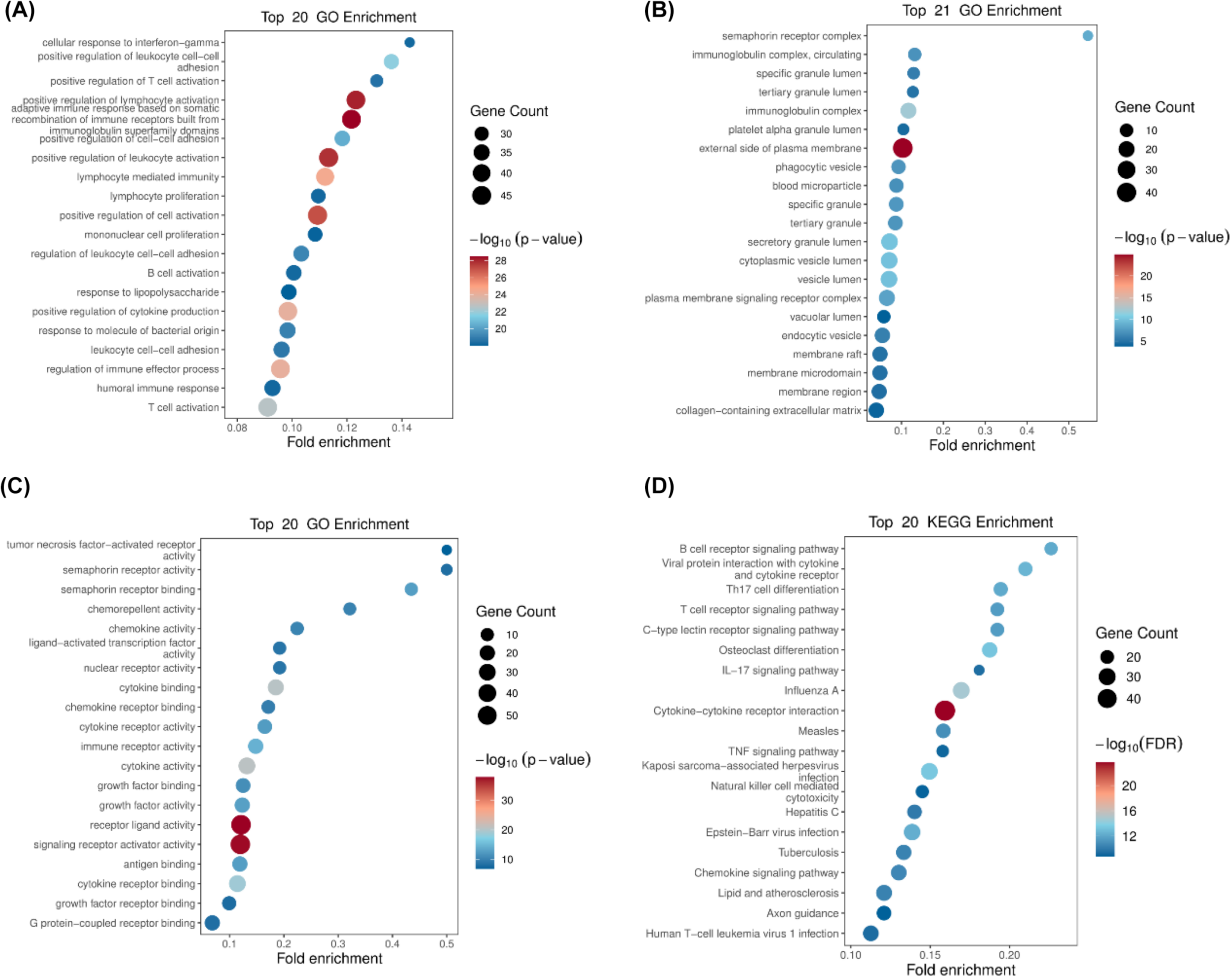
Gene Ontology and KEGG pathway enrichment analysis of DEIRGs. (A) GO analysis for biological process (BP), (B) cellular component (CC), (C) molecular function (MF) of DEIRGs in CST treated LPS‐activated PBMCs. (D) KEGG pathway enrichment of DEIRGs. Pathway enrichment analysis revealed the top 20 enrichment pathways in CST treated LPS‐activated PBMCs. Fold enrichment was exhibited in the X‐axis and different categories are shown in the Y‐axis. The number of genes enriched in particular category are manifested by the size of the circle. The colour of the circle denotes different significance.

To further explore the enriched pathways and functions of the DEIRGs, the genes were performed Gene ontology and KEGG pathway enrichment analysis. These genes are mainly positive regulation of lymphocyte activation and leukocyte activation, located in external side of plasma membrane, and mainly exhibit receptor ligand activity and signalling receptor activator activity (Figure 7A–C). And the KEGG enrichment analysis showed the top 20 significant pathways, including cytokine−cytokine receptor interaction, C‐type lectin receptor signalling pathway, IL‐17 signalling pathway, natural killer cell‐mediated cytotoxicity, chemokine signalling pathway, B cell and T cell receptor signalling pathway. Some viral infectious diseases such as Influenza A, Kaposi sarcoma−associated herpesvirus infection, Epstein–Barr virus infection and Human T‐cell leukaemia virus 1 infection were detected. Furthermore, some enriched DEIRGs were associated with apoptotic signalling, such as the TNF signalling pathway (Figure 7D).

## 4 DISCUSSION

Rising rate of antibiotic therapy failure and advances in immunomodulatory therapy have inspired a new horizon for non‐traditional antibacterial therapeutic in AMR.^22^, ^31^ LPS, also known as endotoxin, is a component of the cellular wall of Gram‐negative bacteria and is also a most potent mediator of all the microbial which is participated in the pathogenesis of severe sepsis and septic shock.^32^ We used the in vitro inflammatory model induced by LPS to investigate whether and how CST possessed immunoregulation properties in response to bacterial pathogens. Data from our study demonstrated that CST promoted the fluorescent latex beads to be up‐taken and modulated the gene expression and protein levels of inflammatory cytokines in PBMCs. Although it had a certain pro‐inflammatory effect through increasing the expression level of TNF‐α in the early stage, CST overall appears strong anti‐inflammatory properties through reducing the expression levels of pro‐inflammatory cytokines (IL‐1β, IL‐6 and IFN‐γ) at 6 h and increasing the expression levels of anti‐inflammatory cytokines (IL‐4, IL‐10, IL‐13 and TGF‐β1) at 24 h. The double regulatory effect of CST may be beneficial for host to clear pathogenic microorganisms, reduce tissue injury in the acute phase of inflammation and facilitate tissue repair at the late stage of inflammation. Notably, in the presence of LPS, CST had the favourable immunomodulatory effect regardless of with or without pre‐incubation with PBMCs, and the latter (without pre‐incubation with PBMCs) performed better. The superior effect might be partly explained that LPS was bound and/or neutralised by CST, which might be a hypothesis of the immunomodulatory action. The conventional antibiotic treatments usually lead to bacteria death without LPS neutralisation, which was mainly responsible for severe inflammation. The considerable increased findings recent justified the strategies to block and/or neutralise endotoxins for severe inflammation.^33^

CST didn't decrease the secretion of TNF‐α induced by LPS. Instead, it might maintain the gene and protein expression level of TNF‐α through CLRs and natural killer cell‐mediated cytotoxicity (Figure 7D). These pathways played the essential role in innate immunity, which demonstrated the host‐protective effect of CST. Besides, CST promoted the release of CCL24, which was a chemokine (also called chemotactic cytokine) (Figure 5D, Table 2). Chemokines directed the migration and localization of almost all immune cell, and they were appreciated as important mediators for not only acute inflammation but also the generation of humoral and adaptive cellular immune responses.^34^, ^35^, ^36^, ^37^ It was also reported that CCL24 showed potent antimicrobial activity on *S. pneumoniae*, *S. aureus*, Non‐typeable *H. influenzae* and *P. aeruginosa*.^38^ Furthermore, FPR3 (Figure 5D, Table 2), significantly upregulated by CST, was a receptor of formylpeptides (derived from PAMP or DAMP). FPR3 could specifically recognise formylpeptides from invading pathogens or damaged host mitochondria and play a crucial role in host innate immune defence and inflammation.^39^, ^40^, ^41^, ^42^ SPP1, also known as osteopontin, is one of the important macrophage cytokines associated with immune cell activation, lysosomal activity and phagocytosis.^43^, ^44^, ^45^, ^46^ The promoted phagocytosis in our study (Figure 1, Figure 7D) may be partly related to the increased secretion of SPP1 (Figure 5D, Table 2). These early inflammatory responses contributed to the rapid elimination of pathogens and rapid signal transmission about the type and severity of the infection to the host so that appropriate immune response could be further arranged.

However, excessively activated innate immune response to infection could also lead to a series of cell/tissue damage and molecular disorders, resulting in life‐threatening organ dysfunction and failure.^47^, ^48^ Our data showed that 10 μg/mL CST reduced LPS‐induced pro‐inflammatory cytokine release of PBMCs including IL‐1β and IL‐6, which was consistent with previous report on macrophages from rats, as well as the decreased production of IFN‐γ in our study. In addition, CST also greatly promoted the apoptosis‐related signalling (not shown, padj = 0.000545), such as the TNF signalling pathway which DEIRGs were enriched in (Figure 7D). Apoptosis is an active non‐inflammatory programmed cell death used by phagocytes as one of the classic strategies to clear intracellular pathogens, which played essential roles in the control of the immune response.^49^, ^50^ Afterwards, cell fragments (apoptotic bodies) were almost inevitably and rapidly phagocytosed by phagocytes and effectively inhibited pathogens colonization, replication and dissemination.^51^ Indeed, the efficient clearance of apoptotic cells could actively prevent apoptotic cells production of excessive pro‐inflammatory cytokines and facilitate the maturation of dendritic cells and antigen presentation by antigen‐presenting cell (APC), which contributed to the maintenance of normal cellular homeostasis tissue repair.^52^, ^53^ Moreover, apoptosis followed by rapid uptake into adjacent phagocytic cells such as macrophages or dendritic cells could also evoke an anti‐inflammatory signalling through increasing anti‐inflammatory cytokine release such as IL‐4, IL‐10, IL‐13 and TGF‐β1, which were consistent with our study results.^54^ Additionally, in the presence of LPS, CST downregulated some T cell receptors (TCRs) complex involved in adaptive immune response, such as TRBJ2‐3, TRBJ2‐4, TRBJ2‐7, PAK4 and DLL4 (Figure 5D, Table 2). T cell activation requires TCRs ligation, but overactive T cells could continually aggravate inflammatory response and result in organ dysfunction.^55^, ^56^, ^57^ And the balance of the T‐cell‐mediated host immune response is a major determinant of outcome.

Consistent with previous animal study, CST significantly modulated the cytokines expression levels (TNF‐α, IL‐6 and IL‐1β), which appeared to involve in conserved MAPK pathway (not shown, padj = 3.85E‐07).^29^, ^30^ Actually, our KEGG pathway enrichment analysis showed that more complex acquired immunity and inflammatory response processes were highly enriched by the DEIRGs including B cell and T cell receptor signalling pathway, IL‐17 signalling pathway, cytokine−cytokine receptor interaction and viral infections (Figure 7D). And the cytokine‐cytokine receptor interaction was the most significantly enriched by DEIRGs, which may summarize our qRT‐PCR and ELISA results above (Figures 2 and 3). Cytokine‐receptor complexes were protein–protein interactions and exerted vast immunoregulatory effects on host. Based on the tremendous importance of these interactions in health and disease, finding and developing drugs as cytokine agonists and antagonists could provide potential and novel therapies to varieties of diseases including immune disorder, cancer and infection.^58^ While cytokine‐related drugs were mostly macromolecular proteins,^59^ several recent reports about small‐molecule drugs in protein–protein interactions showed great appeal,^60^ which may be a novel direction for CST study.

## 5 CONCLUSIONS

Taken together, present study showed the anti‐inflammatory and immunoregulatory effects of CST in LPS‐induced PBMCs, which could reset the inflammatory response. It required further studies to determine the immunomodulation effect of CST in vivo in order to expand its usage as a therapeutic strategy in immune dysregulated diseases.

## AUTHOR CONTRIBUTIONS

**Huiling Chen:** Writing – original draft (lead). **Tianli Yang:** Data curation (equal). **Yiran Xu:** Writing – review and editing (lead). **Beibei Liang:** Validation (equal). **Xianyong Liu:** Supervision (equal). **Yun Cai:** Conceptualization (lead).

## FUNDING INFORMATION

This work was supported by the National Natural Science Foundations of China [grant number 82073894]; Cultivation Project of PLA General Hospital for Distinguished Young Scientists [grant number 2020‐JQPY‐004]; and New Medicine Clinical Research Fund [grant number 4246Z512].

## CONFLICT OF INTEREST STATEMENT

The authors confirm that there are no conflicts of interest.

## Data Availability

All date included in this study are available upon request by contact with the corresponding author.
